# A Bayesian hierarchical gene model on latent genotypes for genome-wide association studies

**DOI:** 10.1186/1753-6561-8-S1-S45

**Published:** 2014-06-17

**Authors:** Ian Johnston, Luis E Carvalho

**Affiliations:** 1 Mathematics and Statistics Department, Boston University, 111 Cummington Mall, Boston, MA 02215, USA

## Abstract

The primary goal of genome-wide association studies is to determine which genetic markers are associated with genetic traits, most commonly human diseases. As a result of the "large *p*, small *n*" nature of genome-wide association study data sets, and especially because of the collinearity due to linkage disequilibrium, multivariate regression results in an ill-posed problem. To overcome these obstacles, we propose preprocessing single-nucleotide polymorphisms to adjust for linkage disequilibrium, and a novel Bayesian statistical model that exploits a hierarchical structure between single-nucleotide polymorphisms and genes. We obtain posterior samples using a hybrid Metropolis-within-Gibbs sampler, and further conduct inference on single-nucleotide polymorphism and gene associations using centroid estimation. Finally, we illustrate the proposed model and estimation procedure and discuss results obtained on the data provided for the Genetic Analysis Workshop 18.

## Background

In genome-wide association studies (GWAS), we infer which single-nucleotide polymorphisms (SNPs) are associated with a trait. We cast this problem as variable selection; however, because the number of observations in a GWAS data set, *n*, is typically much smaller than the number of SNPs, *p*, this is a "large *p*, small *n*" problem [1]. This problem is aggravated by the computational cost of trying to fit a complex statistical model involving hundreds of thousands of SNPs. As a result, few publications have incorporated interaction testing of GWAS data [2]. Models that have been proposed include, but are not limited to, simple logistic regression models that only look for marginal effects [3], more complicated logistic regression models that allow for interactions [4], and nonlinear models [5]. Bayesian models have also been explored as an effective way to reduce the curse of dimensionality (eg, Ref. [6] and references therein). Our objective is to supplement these models with one that accounts for correlation in the model specification and that can exploit SNP groupings within genes.

## Methods

### Latent genotypes

It is usual to assume that the genotype data *X *is known as observed data and to define the likelihood of the trait response *y *conditional on *X*. This can be problematic for inference because *X *depends on minor allele frequencies (MAFs), and elements of *X *can be highly correlated as a result of linkage disequilibrium (LD). It is possible to simulate genotypes by sampling and dichotomizing a random vector from a multivariate normal distribution with a zero mean vector and a covariance matrix that can be computed from the correlation between SNPs [7]. We propose modeling *X *as though it was generated in this way; that is, we observe, in *X*, a correlated and categorized--by allele frequencies--version of the latent genotypes, which we denote *Z*. We model *y *using *Z *in place of *X*.

### Approximation

Instead of obtaining latent genotypes for each marker and individual, we settle with an approximation that allows us to fit a model with many SNPs. Denoting the continuous but correlated genotypes by *U*, we compute ${\hat{U}}_{ij}=E\left[U_{ij}|X_{ij}\right]$, and then, ${\hat{Z}}_{i}=C^{-1}{\hat{U}}_{i}$, where *C *is the correlation matrix. For now, *C *is estimated using the sample correlation matrix.

### Hierarchical gene model

We assume that *y *is quantitative and depends on *Z *and covariates *V *through a linear expectation:

$$y_{i}|z_{i},v_{i},\beta ,\eta ,\tau^{2}\sim \text{ ind}\text{. Normal}\left(v_{i}^{T}\eta +z_{i}^{T}\beta ,\tau^{2}\right),\;i=1,\;...,\;n$$

We define $\theta_{j}\in \left\{0,\;1\right\}$ to indicate if the *j*^th ^marker is associated with the trait and want to use the posterior distribution of each $\theta_{j}$ to make inference on which markers are most likely to be associated with the trait. Using *θ*, we define a spike-and-slab prior on *β*, $\beta_{j}|\theta_{j}\sim \text{ ind}\text{. }\theta_{j}\text{Normal}\left(\text{0,}\sigma^{\text{2}}\right)+\left(1-\theta_{j}\right)\delta_{0}\left(\cdot \right)$, where $\delta_{0}\left(\cdot \right)$ is the Dirac delta function at zero [8]. We use $\text{Normal}\left(0,\;\sigma^{2}\right)$ as a prior for *η*, and integrate out *β *and *η *to obtain a simpler likelihood:

$$y|Z,\;\theta \sim \text{Normal}\left(0,\;\tau^{2}I_{n}+\sigma^{2}VV^{T}+\sigma^{2}Z\text{ Diag}\left(\theta \right)Z^{T}\right)$$

We are also interested in possible effects on SNPs as a result of proximity to genes. These effects can be captured in our model by embedding a *hierarchy*: if $\gamma_{g}$ is an indicator for gene *g *being *active*, then we give a positive or negative boost to the probability that a SNP is associated based on the number of active genes that cover it. We define random parameters $\xi_{0}$, which indirectly defines the prior probability for any SNP to be associated with the trait, and $\xi_{1}$, which accounts for a boosting effect, and write the hierarchy as follows:

$$\theta_{j}|\gamma \sim \text{Bernoulli}\left[\text{logi}{\text{t}}^{-1}\left(\xi_{0}+\xi_{1}\underset{g\in j}{\sum }\gamma_{g}^{*}\;/\;n_{j}\right)\right]$$

where $\gamma_{g}|\alpha \sim \text{Bernoulli}\left(\alpha \right)$, $\gamma_{g}^{*}=2\gamma_{g}-1$, $n_{j}$ is the number of genes that cover $\theta_{j}$, and *α *is the prior probability of a gene being active. To sample *θ *and *γ *from their posterior distributions, we adopt a Gibbs sampling procedure with Metropolis-Hastings steps to sample from the posterior distributions of $\xi_{0}$, $\xi_{1}$, and *α*. After checking for convergence, we use the centroid estimator to estimate the posterior probability of association (PPA) of the *j*^th ^SNP, $\psi_{j}$, based on *N *samples from this procedure as ${\hat{\psi }}_{j}=\hat{\text{P}}\left(\theta_{j}=1|y,Z\right)=\overset{N}{\underset{s=1}{\sum }}\theta_{j}^{\left(s\right)}/N$, and similarly for $\text{P}\left(\gamma_{g}=1|y,Z\right)$, for each gene *g*. By increasing the "boost" parameter $\xi_{1}$, we can place more weight on the information from the gene level. This regularizes the SNPs such that by tuning $\xi_{1}$ we may adjust the PPA level of separation between causal and noncausal SNPs.

### Centroid estimator

An ubiquitous estimator in Bayesian inference is the maximum *a posteriori *(MAP) estimator, ${\hat{\theta }}_{M}=\underset{\theta \in {\left\{0,1\right\}}^{p}}{\text{argmax}}\text{ P}\left(\theta |y,Z\right)$, but ${\hat{\theta }}_{M}$ may correspond to a sharp peak in a multimodal and structured posterior space that does not gather much posterior mass around it. An estimator that is arguably better suited for complex spaces is the centroid estimator, ${\hat{\theta }}_{C}=\underset{\tilde{\theta }\in {\left\{0,1\right\}}^{p}}{\text{argmax}}\;{\text{E}}_{\theta \text{|y,X}}\left[H\left(\theta ,\tilde{\theta }\right)\right]$, where $H\left(\cdot ,\cdot \right)$ is Hamming distance. For unconstrained spaces such as ours, it can be shown that ${\hat{\theta }}_{C}$ is a consensus estimator; that is, ${\left({\hat{\theta }}_{C}\right)}_{j}=I\left[\text{P}\left(\theta_{j}=1|y,Z\right)>0.5\right]$. The centroid estimator can be shown to be closer to the mean than to a mode of the posterior space of SNP associations, and so offers a better summary of the posterior distribution of *θ*[9].

## Results

**Figure 1:**
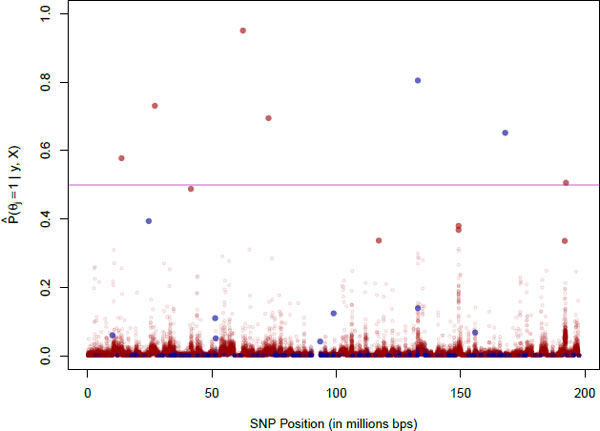
**Posterior probability of association (PPA) of SNPs on chromosome 3**. The top 10 highest PPA have opaque dots (genotypes: raw in red, latent in blue).

**Figure 2:**
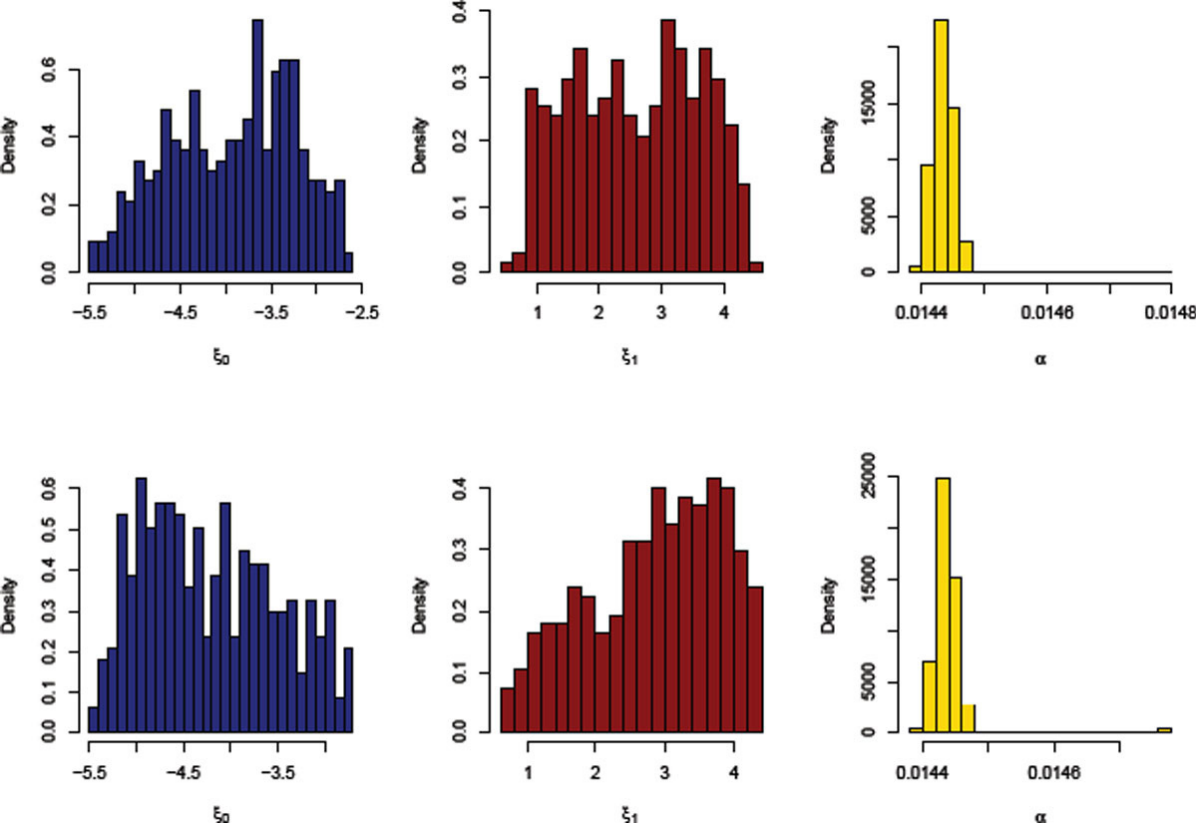
**Expected values of the posterior distributions of **$\xi_{0}$, $\xi_{1}$, and *α*. Histograms of estimates across all windows (genotypes: raw on top, latent on bottom).

Using the GWAS data set provided for Genetic Analysis Workshop 18 (GAW18), we modeled the first systolic blood pressure measurements as *y*, treated the 64,780 SNPs on chromosome 3 with MAF >0 as *X*, and an intercept term and sex as *V*. After eliminating individuals with missing data, 132 *unrelated *individuals remained. As only the real phenotypes were used, the analysis was performed without any knowledge of a simulating model. To run the model efficiently, we constructed 336 blocks such that the breaking points were positioned where adjacent SNPs had distance greater than 15,000 kilobases (kb). After a prior sensitivity analysis, we set $\tau^{2}=300$ to avoid selecting too many or too few SNPs. We set the hyperparameters for $\xi_{0}$ and $\xi_{1}$ such that they had prior distributions of Uniform (−6, −2) and Uniform (0, 5), respectively, and assigned a prior beta distribution to *α *with stringent hyperparameters so as to concentrate the probability distribution about a low expected value of around 0.015, which corresponds to expecting 5 blocks out of 336 total to have 1 active gene. Table 1 presents the 5 SNPs with the largest estimates of the PPA for both raw and latent genotypes, and Figure 1 depicts the results of the centroid estimator. The red dots (raw genotypes) in Figure 1 follow a pattern similar to *p *values in Manhattan plots; a SNP with a high PPA is surrounded by SNPs with relatively higher PPA. The blue dots, on the other hand, do not show this pattern because the latent genotypes have been decorrelated. Moreover, we observe that 90.4% of the SNPs have a latent genotype PPA smaller than their raw genotype PPA. Figure 2 shows histograms of the estimated expected values of the posterior distributions of $\xi_{0}$, $\xi_{1}$, and *α*. The positive effect of using the latent genotypes as indicated by the smaller values of $\xi_{0}$ and the larger values of $\xi_{1}$ is that, *a priori*, the SNPs have a lower PPA, and so gene effects are more cleanly observed. When using the raw genotypes, the SNP with the highest PPA is intronic to the *CADPS *gene. This gene interacts with the *DRD2 *gene, which is related to the negative regulation of blood pressure [10]. We observe another SNP intronic to a gene, *FBLN2*, that may also be involved in the regulation of blood pressure [11]. The latent genotypes with PPA above 0.5 are not located in any genes with a known connection to blood pressure.

**Table 1:** Top 5 SNPs for original raw (normal text) and latent genotypes (bold)

SNP	Position	MAF	SNP PPA	Gene	Gene PPA
rs17688430	62458083	0.16	0.95	*CADPS*	0.012
rs7616789	27024158	0.23	0.73	*-- *	--
rs1565471	72736592	0.43	0.70	*--*	--
rs3773282	13630307	0.29	0.58	*FBLN2*	0.006
rs13068005	192388678	0.47	0.50	*FGF12*	0.022
**rs10935047**	**132815378**	**0.38**	**0.80**	***TMEM108***	**0.016**
**rs9872284**	**167951681**	**0.03**	**0.65**	***--***	**--**
**rs3856621**	**24566228**	**0.40**	**0.39**	***--***	**--**
**rs7631163**	**132837961**	**0.44**	**0.14**	***TMEM108***	**0.016**
**rs774952**	**98919271**	**0.04**	**0.12**	***--***	**--**

## Conclusions

We presented a Bayesian variable selection approach that performs joint inference for quantitative trait association on collections of genetic markers while formally modeling gene effects through a hierarchical influence. In addition, we prescribe centroid estimators that are based on posterior probabilities of association and thus enable a direct interpretation of their values uniformly across studies without having to correct for multiple testing. We also proposed the novel use of latent genotypes as a way to account for SNP correlations caused by LD. We believe that this method offers a reasonably accurate and flexible assumption because genotypes are corrected directly in the model instead of considered in the estimation procedure, as, for example, as kernel weights in the sequence kernel association test (SKAT) [12]. However, unfortunately, we were not able to find meaningful effects in the GAW18 data set when using latent genotypes that would point to interesting genes. This outcome can be explained by many factors, including a low sample size, an inaccurate representation of the correlation across markers, and a poor choice of SNP blocks, and thus warrants further investigation. Moreover, a more thorough prior sensitivity analysis would recommend a less stringent distribution for some hyperparameters, mainly *α*, that would favor more genes to be active.

## Competing interests

The authors declare that they have no competing interests.

## Authors' contributions

IJ implemented the sampling algorithm in C++ and prepared the results; LEC proofread and approved the final manuscript.
